# Acceptability and Preliminary Efficacy of a Novel Web-Based Physical Activity for the Heart (PATH) Intervention Designed to Promote Physical Activity in Adults With Obesity: Protocol for a Pilot Randomized Controlled Trial

**DOI:** 10.2196/67972

**Published:** 2025-03-18

**Authors:** Jacob Kariuki, Lora Burke, Kirk Erickson, Susan Sereika, Sudeshna Paul, Jessica Cheng, Heran Biza, Amjad Abdirahman, Katherine Wilbraham, Heather Milton, Cornelius Brown, Matthew Sells, Foster Osei Baah, Jessica Wells, Rasheeta Chandler, Bethany Barone Gibbs

**Affiliations:** 1 Emory University Atlanta, GA United States; 2 School of Nursing University of Pittsburgh Pittsburgh, PA United States; 3 Neuroscience AdventHealth Research Institute Orlando, FL United States; 4 T. H. Chan School of Public Health Massachusetts General Hospital Harvard University Boston, MA United States; 5 NYU Langone Health New York University New York, NY United States; 6 Department of Epidemiology and Biostatistics School of Public Health West Virginia University School of Public Health Morgantown, WV United States

**Keywords:** obesity, physical activity, cardiometabolic risk, body positivity, cardiovascular fitness, self-efficacy

## Abstract

**Background:**

Even in the absence of weight loss, any level of physical activity (PA) can reduce the risk of cardiovascular disease among individuals with obesity. However, these individuals face multifaceted barriers that reduce their motivation and engagement in PA. They prefer programs that are convenient, fun to engage in, and feature people who they can relate to. Yet, there is a paucity of PA interventions that are designed to incorporate these preferences. We designed the web-based PA for The Heart (PATH) intervention to address this gap.

**Objective:**

This study aimed to describe the protocol of a study that aims to examine the acceptability and preliminary efficacy of PATH intervention among insufficiently active adults with obesity aged at least 18 years.

**Methods:**

This is a 6-month pilot randomized controlled trial (RCT), using a parallel design with 1:1 allocation to intervention or control group. The PATH intervention group is given access to the PATH platform, but the resources each participant can access are tailored according to their baseline fitness level. Control group receives a self-help PA handout. Both groups self-monitor their PA using Fitbit (Google) and have Zoom (Zoom Video Communications) meetings twice a month with either the health coach (intervention) or study coordinator (control). The outcomes at 6-months include acceptability, changes in PA, and cardiometabolic risk from baseline to 6-months.

**Results:**

We screened 763 individuals for eligibility and 89 participants were enrolled and randomized to the intervention (45/504, 50.6%) and control arms (44/504, 49.4%). The average age was 48.7 (SD 12.17) years, and most participants were female (81/504, 90.1%), Black (45/504, 50.6%), and non-Hispanic (83/504, 93.3%). No systematic differences in baseline characteristics were observed between the study arms. The 6-month intervention is currently underway, and the completion of follow-up data collection is expected in February 2025, with results to be published soon after.

**Conclusions:**

The PATH intervention offers a promising, evidence-based approach to overcoming the barriers that have hindered previous PA programs for adults with obesity. It can support new and existing programs to foster long-term maintenance of health-enhancing PA.

**Trial Registration:**

ClinicalTrials.gov NCT05803304; https://clinicaltrials.gov/study/NCT05803304

**International Registered Report Identifier (IRRID):**

DERR1-10.2196/67972

## Introduction

Only about 14% of adults with obesity attain the minimum levels of physical activity (PA) recommended by public health guidelines to achieve health benefits [1-3].Low levels of PA contribute to the high population burden of cardiovascular disease (CVD) as they increase the relative risk of stroke, coronary heart disease, and diabetes by 60%, 45%, and 30%, respectively [4-6]. Even in the absence of weight loss, regular PA can significantly reduce the risk of CVD among individuals with obesity [3,7,8]. However, individuals face complex and multifaceted barriers that reduce their motivation and engagement in regular PA for meeting current recommendations [9,10].

Barriers to PA associated with obesity include stigma, shame, poor fitness, and low self-efficacy. These evoke fears of embarrassment and pain, contributing to aversion and avoidance of PA [10-12]. To mitigate these barriers, web-based PA programs targeting adults with obesity have been developed. Preliminary data suggest improved retention for online programs, but the effects on PA are heterogeneous [13]. Limitations of these interventions include the absence of human contact, “one-size-fits-all” strategies, unmet weight-loss expectations, and generic content that fails to address barriers associated with obesity [10,14]. We [15] and others [9,10] have reported that individuals with obesity prefer programs that are convenient, fun to engage in, and feature people who they can relate to with respect to body size, fitness level, and age. Yet, there is a paucity of web-based PA programs that are intentionally designed to flexibly incorporate these preferences [10,16,17].

To address the limitations of previous interventions, our research team designed the web-based PA for The Heart (PATH) intervention. PATH leverages openly accessible platforms, such as YouTube (Alphabet Inc), to provide workout videos that match the specific preferences expressed in our formative studies and the extant literature. In developing PATH, we used an iterative bottom-up approach where our target population was engaged in the selection and rating of the workout videos. Then, highly rated workouts (≥3.5/5 stars) were vetted by the study team for content relevance and safety and then curated on our PATH website in 3 intensity levels (beginner, intermediate, and proficient) to foster gradual progression from low- to high-intensity PA. We added backend features that enable a remote health coach to help users set their PA goals and select a PA regimen that is safe for their fitness level. Each PATH user has a personalized dashboard displaying their recommended workouts and progress toward their PA goals.

We have successfully beta-tested the PATH platform (N=25) and completed a 12-week feasibility study where we met our recruitment goal (N=82) and attained excellent retention (96%). Intervention engagement was high, and the PATH group significantly increased objective moderate to vigorous PA (MVPA) indicating preliminary efficacy [18]. The feedback we obtained from this study included the need to provide resources that can help participants improve their diet, provide feedback on cardiometabolic health indicators, and the need to improve our machine learning algorithm designed to keep the PATH platform up to date.

We used this feedback to further refine the PATH platform by curating nutritional resources focusing on improving diet quality and added a user-facing dashboard with feedback on 5 measures of cardiovascular health including physical activity, blood pressure (BP), weight, heart rate, and sleep efficiency. Also, we optimized the platform’s machine learning algorithm (PATH Fresher) that continually identifies new workout videos that match those preferred by the PATH users to be used in future updates of the platform.

This study describes the latest protocol (version #7, 12/16/2024) of a 6-month pilot randomized controlled trial (RCT), using a parallel design with 1:1 allocation to intervention or control, and designed to assess the preliminary efficacy of the optimized PATH intervention for promoting adherence to PA guidelines among 88 insufficiently active adults with obesity. We hypothesized that participants who are randomized to the optimized PATH intervention will show greater increases in PA at 6 months compared with those assigned to the attention control group. We also hypothesized that the optimized PATH intervention group will have a more favorable cardiometabolic risk profile at 6 months compared with the attention control group.

## Methods

### Study Design

This is a 6-month, ongoing parallel group RCT including 89 participants with insufficient activity and obesity who are randomized 1:1 to either the PATH intervention or an attention control group. The PATH intervention group are given access to the PATH platform, but the resources each participant can access are tailored according to their baseline fitness level (details described below under optimized PATH intervention). In addition, the intervention group receive text and or email reminders to log into PATH and do their workouts based on their preferred schedule and will have twice per month online meetings with fitness coaches to monitor progress and review PA goals. The control group receives the Be Active Your Way booklet which is an evidence-based self-help resource for promoting PA [19] and twice per monthly online meetings with the study coordinator to check-in on their progress in the study and maintain contact. The staff members conducting the study assessments are blinded to randomized group allocation.

### Setting and Participants

The study is conducted at Emory University, with participants recruited primarily from the Atlanta metro region. Eligibility criteria include reliable access to the internet, age ≥18 years, BMI ≥30 kg/m^2^, successful self-monitoring of PA (≥4 days with ≥10 hours wear time) via a waist-worn ActiGraph accelerometer during a run-in period, and classification as insufficiently active according to the PA Guidelines (<150 min of MVPA per week) based on self-reported PA from the Behavioral Risk Factor Surveillance System PA questionnaire [20]. Exclusion criteria include current participation in a lifestyle modification or weight loss study, pregnancy or intention to become pregnant within 6 months, mobility restrictions, use of implantable electronic medical devices, or any condition that requires supervised PA (eg, stroke). Those with any condition that the PA Readiness Questionnaire (PAR-Q+) [21] identifies as requiring primary care provider review before engaging in unsupervised PA will be required to obtain medical clearance before they are enrolled in the study.

### Recruitment

#### Overview

To obtain our target sample (N=89), we leverage resources available to Emory investigators, including Georgia Clinical and Translational Science Institute, Research Match (registry of research volunteers), electronic mailings, announcements on social media sites, and fliers posted in the community. We are also recruiting from primary care practices affiliated with Emory Health care, with an aim to recruit a diverse sample that includes ≥25% men and ≥30% racial and ethnic minorities.

#### Screening and Online Questionnaires

Individuals who respond to recruitment solicitations are directed to a web link that provides a brief overview of the study and eligibility criteria. Those who are eligible are guided to follow a link to complete 9 brief questionnaires which are outlined with their validation references in Table 1. For participant convenience, the questionnaires are administered via the REDCap (Research Electronic Data Capture; Vanderbilt University) platform so that they can be completed online in one or multiple sittings within 30 minutes. Data from the questionnaires are used for eligibility screening and will eventually allow us to examine the impact of sleep disturbance, health status, stress, and risk of depression on study outcomes. The eligibility criteria are embedded within the online surveys so that the data collection is terminated when a potential participant enters information that makes them ineligible (eg, history of stroke). Those who complete the REDCap questionnaires and remain eligible for the study are scheduled for a 20-minute phone interview, during which the Mediterranean Eating Pattern for Americans [22] screener and the PA questionnaire [20] are administered by an interviewer. Those who do not meet current PA guidelines (<150 min of MVPA per week) are scheduled for a 15-minute phone call to set up the technology that they will be using in the study.

**Table 1:** The questionnaires and assessment schedule.

Questionnaires and scheduled assessments			Baseline				6 months		
**Screening, health history, and lifestyle questionnaires**									
	Sociodemographic and medical history questionnaires	✓				✓			
	PA^a^ readiness questionnaire (PAR-Q+) [21]	✓				✓			
	Poffenbarger exercise habits questionnaire [23]	✓				✓			
	Barriers Self Efficacy Scale [24]	✓				✓			
	PROMIS SF^b^ sleep disturbance questionnaire [25]	✓				✓			
	PROMIS SF sleep-related impairment questionnaire [25]	✓				✓			
	Center for Epidemiologic Studies Depression Scale (CES-D) [26]	✓				✓			
	NIH^c^ toolbox perceived stress [27]	✓				✓			
	EQ-5D health status instrument (EQ-5D-5L) [28]	✓				✓			
**Questionnaires measuring PA and potential PA mediators**									
	Mediterranean eating pattern for Americans questionnaire [22]			✓				✓	
	BRFSS^d^ physical activity questionnaire [20]			✓				✓	
	All of us research program lifestyle survey (smoking and alcohol)			✓				✓	
	Exercise Self-efficacy Scale [29]			✓				✓	
	Self-regulation Questionnaire [30]			✓				✓	
	Physical Activity Enjoyment Scale [31]			✓				✓	
	Social Support for Exercise Scale (SSES) [32]			✓				✓	
	Multidimensional Outcome Expectations for Exercise Scale [33]			✓				✓	
**Medication history and cardiometabolic assessments**									
	Medication history				✓				✓
	Zoom supervised self-administered blood pressure				✓				✓
	Zoom supervised self-administered weight	✓				✓			
	Zoom guided self-administered waist circumference	✓				✓			
	Self-reported data for PA index (frequency, intensity, and duration) [34]	✓				✓			
	Zoom supervised dry blood spot sample collection for HbA1C^e^, adiponectin, and lipids (total, LDL^f^, and HDL^g^ cholesterol)	✓				✓			
	CVD^h^ risk score computed using ASCVD^i^ risk calculator [35]	✓				✓			
	Heart health score computed using life’s essential data	✓				✓			
	American diabetes association risk score [36]	✓				✓			
	Fitbit charge 5 data on PA (continuous)	✓				✓			
	ActiGraph GT3X accelerometer data on MVPA^j^ (7 days)	✓				✓			
**Users’ feedback**									
	System Usability Scale [37] and post-intervention survey					✓			

^a^PA: physical activity.

^b^PROMIS SF: Patient-Reported Outcomes Measurements Information System.

^c^NIH: National Institutes of Health.

^d^BRFSS: Behavioral Risk Factors Surveillance System.

^e^HbA_1c_: hemoglobin A_1c_.

^f^LDL: low-density lipoprotein.

^g^HDL: high-density lipoprotein.

^h^CVD: cardiovascular disease.

^i^ASCVD: atherosclerotic cardiovascular disease.

^j^MVPA: moderate to vigorous PA.

#### Technology Set-Up, Informed Consent, and Mediation

Before the technology set-up phone call, we provide potential participants with email instructions on how to install Zoom (Zoom Video Communications; for assessments and coaching), Withings (weight, BP), and Fitbit (PA) apps. During the phone call, the study staff members verified the software installation and provided login credentials for the apps. Next, study staff members review the study procedures and those still interested in the study receive a copy of the informed consent to review and sign via REDCap. Those who consent are asked to complete the All of Us Research Program Lifestyle Survey as well as questionnaires assessing exercise self-efficacy [29], self-regulation [30], PA enjoyment [31], social support [32], and outcome expectancy [33] (Table 1). These validated questionnaires are frequently used to identify facilitators and barriers [29-33] that are known to influence PA and cardiovascular outcomes [38-41]. These questionnaires can be completed in REDCap within approximately 30 minutes and will be repeated at the 6-month follow-up visit. An optional Adverse Childhood Experiences questionnaire will be used to explore difficult childhood experiences that may impact variables of interest in the study. The participants will be asked by the study staff members to give their verbal consent to receive this questionnaire at the end of the study assessment.

#### Baseline Assessment Via Zoom

Participants who meet all eligibility criteria during screening are scheduled for a baseline assessment via Zoom. Before the assessment, each participant will receive a package with all supplies including a Dry Blood Spot Kit, Fitbit Charge 5, ActiGraph GT3X, Withings Arm BP Monitor, Withings Scale, Perfect Waist Tape Measure, and Stretch Band. During the Zoom visit, the study staff members review the data in the PA readiness questionnaire (PAR-Q+) [21], to ensure that individuals who need primary care practitioner clearance obtain it before they are randomized to the intervention or attention control condition. We use the HIPAA (Health Insurance Portability and Accountability Act)-compliant online Fax (SRFax, Ziff Davis Inc) to send and receive faxes on behalf of participants.

The assessments commence with the study staff members reviewing the waist circumference self-measurement video and guidelines developed by the International Chair on Cardiometabolic Risk [42]. The staff members instruct the participants to go to a private location where they can measure their waist on bare skin using the Perfect Tape Measure. The participant shows the locked tape measure to staff members via the camera. There is evidence for strong correlation coefficients (0.8 to 0.9) between self and technician-measured waist circumference using this method [43-47]. Next, the staff members instruct the participant to wear light clothing and stand on the smart scale footpads with their bare feet to measure weight, percentage body fat, percentage body water, muscle, and BMI. The study team obtains these device-recorded measures via the Withings application programming interface (API) with the PATH platform.

We use the clinically validated Withings Arm BP Monitor for self-measurement of BP [48]. The smart monitor has a one-touch easy-to-read digital screen and a cuff that fits an arm circumference of 22-42 cm (larger cuffs available on request). Staff members will instruct the participant to apply the cuff on the bare left arm, then sit in a chair with both feet resting flat on the floor, with the back straight and supported. After 5 minutes of rest with the left arm resting on a flat surface at heart level, participants will be asked to turn on the BP machine and take 3 measurements at 1-minute intervals. The study team will immediately access the device-recorded BP via Withings API with the PATH platform. Participants with elevated BP (>130/80 mm Hg) will be referred to their primary care practitioner, with a request to update the study team if treatment is initiated. Those with severe hypertension (BP ≥180/110 mm Hg) will be asked to seek urgent medical attention before re-evaluation of their eligibility.

Blood samples for measurement of adipokines (tumor necrosis factor alpha [TNF-α], monocyte chemoattractant protein 1 [MCP-1], interleukin [IL]-1 beta [IL-1β], and IL-6, leptin, and adiponectin) and lipids (low-density lipoprotein [LDL], high-density lipoprotein [HDL], and total cholesterol) are collected via volumetric absorptive microsampling approach using Mitra dry blood spot kits [49]. Study staff members supervise participants collecting the fingerstick sample following the steps outlined in the sample collection tutorial [50]. The samples are mailed by each participant to the laboratory using the included return address. Once at the laboratory, the samples are stored at –80^o^ C and will be processed at the end of the study using established protocols for analysis [51].

#### Run-In Period

At the end of the baseline assessment, the staff members will instruct participants to wear the ActiGraph GT3X on their waist and Fitbit on their wrist to monitor PA for a 7-day run-in period. The run-in period will help potential participants appreciate study expectations and will provide objective data on baseline MVPA. Individuals will need to wear the Fitbit and ActiGraph GT3X for ≥10 hours on ≥4 days to be eligible in the study [52].

#### Randomization and Orientation

After the successful completion of the run-in period, nonblinded staff members randomize eligible participants with equal allocation (1:1) to either the PATH intervention or the attention control arm using the REDCap randomization software. Group assignments are generated via a REDCap stratified randomization scheme to achieve a balance between the treatment and control groups regarding age (18-45, 46-64, and ≥65 years), sex (male or female), and race (White or racial and ethnic minorities). The Randomization scheme was developed by the study statistician. After randomization, a study staff member meets with each participant on Zoom to orient them to their randomized group resources. If blinded staff members inadvertently become unblinded, the incident will be noted in the study’s log, and they will not conduct end-of-study assessments on the participant. Participants in both groups are instructed to wear Fitbit on their nondominant hand during the study using a 24-hour wear protocol.

### The PATH Intervention

A health coach will provide each participant with a password-protected profile to access the PATH website and a detailed orientation on how to use all the resources included in PATH. In addition, the health coach will meet remotely with each participant twice per month. The PATH intervention provides counsel and guidance to participants in the development of multiple behavior change strategies to promote long-term adherence to the minimum threshold of PA Guidelines (150 MVPA min/week). The Fitbit and Withing APIs are integrated with our PATH platform to enable near-real-time monitoring of the participant’s progress. The interface also enables us to capture longitudinal trends in PA and to display the progress toward their PA goal on each participant’s dashboard. The coach will work with each participant in the treatment group to develop their PA prescription guided by the FITT-VP (frequency, intensity, type, time, volume, and progression) principle [53], which recommends the frequency, intensity, duration, type, volume, and progression of PA. Although there are no established standards on how to increase PA, available evidence suggests that it is safe to increase MVPA by 10 minutes per week [54]. Although the weekly MVPA goal translates to about 1000 steps of moderate activity per week [55], the participants are asked to target 500 additional steps per week to make sure that the goals are safe for everyone, including those with morbid obesity.

The coach works with each participant to develop a tailored plan geared toward increasing MVPA by ~10 minutes per week with each coaching encounter. Given the scheduled 12 coaching encounters during the 6-month study period, participants at all levels of fitness will have a chance to develop a PA regimen that is adherent to the PA Guidelines. Participants with relatively high baseline PA are advised to target the higher threshold of the PA Guidelines (300 MVPA minutes per week). Our PA prescription process begins by identifying a suitable PATH level for each participant based on their estimated cardiorespiratory fitness level, which is estimated using a predicted maximum rate of oxygen consumption during incremental exercise (VO_2_ maximum) [53]. The VO_2_ maximum is predicted using a validated, nonexercise prediction model for peak VO_2_ whose covariates include sex, age, waist circumference, resting heart rate, and PA index [34].

Individuals with a predicted VO_2_ maximum in the ≤35th percentile for their age and gender are considered for assignment to the Beginner PATH level (includes light-intensity PA metabolic equivalents [METs] of <30 METs). Those with VO_2_ maximum above the 35th percentile rank are assigned to the intermediate PATH level, which includes moderate-intensity workouts (3.0-5.9 METs) in addition to the Beginner PATH content. Based on our pilot experience, it is unlikely that a participant will be assigned to begin at the Proficient PATH level which includes vigorous-intensity workout videos (≥6 METs). Rather, participants will be given access to the level based on their rating of perceived exertion (RPE) scores at the intermediate level and coach evaluation. The coach can change the PATH level based on their assessment of the participant’s capabilities during the meetings.

After assigning the PATH fitness level, the health coach guides each participant in selecting their weekly PA goal and helps them start slowly with a plan to establish regular exercise frequencies of 3-5 days per week. The coach also guides participants to select activities with intensity to help them progress along the PA continuum (ie, from inactive to light PA and then MVPA). To foster safety, participants are instructed to use the RPE Scale [56] as a guide for adjusting the intensity of their PA regimen. The scale ranges from 6 (no exertion at all) to 20 (maximal exertion) and is embedded within each workout video on PATH. The coaches will also help participants set up Fitbit safe heart rate zones based on their fitness levels, with a goal of progressing to the Cardio Zone (70% and 84% of maximum heart rate) by the end of the study.

**Figure 1:**
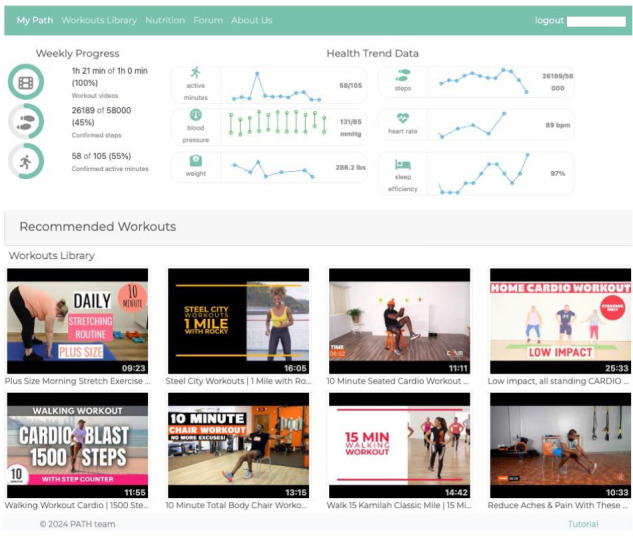
Participants' physical activity for the heart platform dashboard.

Participants are asked to use workouts that elicit perceived exertion ratings between 12 and 14 (ie, moderate intensity) on the RPE Scale. The coach reviews progress made every 2 weeks. More intensity is allowed, including the transition to the next PATH level, when most workouts within the assigned PATH level are perceived to be “fairly light” (≤11 on the RPE Scale). If a participant forgets to rate their workouts, their heart rate during the workout sessions is reviewed during the coaching session, and a goal that aligns with each person’s capabilities is discussed between the health coach and participant. This method ensures the prescribed regimen is based on ability and fitness status. Although the health coach provides examples of workout videos that could help participants attain their personalized goals, each participant has access to all resources within their PATH level and is encouraged to select a regimen that includes their preferred workout videos and other types of PA appropriate for their fitness level. The workouts that are recommended by the health coach and those selected by each participant as favorites are featured on their PATH dashboard alongside the self-monitoring data (Figure 1).

The PA prescription is revised during the online meetings with the coach to reflect the progress made toward the study goal. Those who attain adherence to the PA Guidelines within the first 3 months will achieve the study goal and their plan for the remaining 3 months will be to maintain the improved PA regimen. However, each participant can opt to continue with a gradual increase in PA duration and intensity. To simplify access and support maintenance of PA, the workout videos are organized into categories (eg, walking, dance, and steps aerobics) and can be sorted by duration and METs. To reduce the risk of injury, the amount and intensity of PA are increased gradually, with emphasis on duration followed by intensity per the American College of Sports Medicine [53]. The coach, in consultation with the principal investigator (PI), may discontinue or modify any participant’s PA prescription in response to the potential for harm, participant request, or improving or worsening disease. To complete the PA prescription, participants are asked to suggest their preferred schedule for receiving motivational PA reminders (eg, “Remember to work out on PATH today! Exercise doesn’t have to leave you exhausted for it to make a difference in your health — M. Richardson”). The short motivational messages are randomly selected from a bank with >270 vetted messages. Also, participants are asked to provide a schedule for the Zoom meetings with a health coach to monitor progress and revise PA goals. During the study, the participants are encouraged to share their experiences on the PATH community forum to foster community and social support. An end-of-study survey will capture feedback on participant experiences and the most helpful aspects of the PATH intervention resources.

#### The Control Condition

After randomization, study staff members schedule a Zoom meeting with each control group participant where they are guided to use their tracker and Be Active Your Way Guide [19]. They also schedule a check-in meeting with them every 2 weeks, with the intention to keep the contact between the groups as similar as possible.

#### Protocols for Both Treatment and Control Arms

During the twice per month meetings with intervention and control groups, the participants receive early morning reminders to empty their bladder and step on the scale before meeting with the study staff members who supervise BP measurements on Zoom. These data are used to monitor trends in BP, weight, and body composition during the study period. Both the intervention and control arms receive an email with a brief PDF addressing 1 diet component every 4 weeks. The diet information is also available to them as modules on the PATH platform (control group access on PATH is limited to the nutritional resources). At the end of the 6-month study assessments, both control and intervention group participants are given the option to continue using the PATH platform at their convenience without any interaction with the coaches.

### Study Outcomes

#### Indicators of PATH Fresher Algorithm’s Effectiveness in Identifying Workouts That Match User Preferences

Effectiveness of the PATH Fresher algorithm will be indicated by its ability to analyze PATH users’ data, spin up new browser sessions in the background to activate the YouTube recommender system, and select new workouts like those highly rated (≥3.5/5 stars) by study users. At the end of the study, the coaches will vet the new workouts recommended by the PATH fresher algorithm. The acceptance of ≥50% of the top twenty recommended workouts for inclusion in PATH will indicate the effectiveness of the PATH fresher algorithm. The systems usability survey [37] will be completed by participants to survey satisfaction with the PATH platform.

#### Acceptability of the Disseminated Educational Resources Focusing on Improving Diet Quality

The resources will be curated from an extensive library of standard behavioral treatment content previously developed and validated by the SMARTER study team [57,58]. Their acceptability will be indicated by 70% of the participants rating the materials as helpful via a researcher-developed end-of-study survey (Multimedia Appendix 1).

#### Measures of PA and Adherence to PA Guidelines

The efficacy of PATH in enhancing adherence to PA guidelines will be evaluated at 6 months using MVPA data collected using the Actigraph GT3X device [59] worn on the waist for 7 days at 6 months. Adherence to PA guidelines will be defined as achieving ≥150 minutes of MVPA per week. Percent change in adherence will be calculated as follows: ([postintervention MVPA–baseline MVPA]/recommended MVPA×100). Between-group differences in % change in adherence and the proportion of individuals who attain the recommended MVPA will be evaluated. Established and novel methods will be used to process ActiGraph data which will be considered valid if ≥4 days with ≥10 hours of wear time are measured [59]. ActiGraph GT3X nonwear time was defined using the Choi wear time validation algorithm [60]. Fitbit valid data were indicated by ≥4 days with ≥500 steps per day as recommended by Thorndike et al [61] and Bizhanova et al [62]. Sustained PA engagement will be evaluated via Active Zone Minutes data collected via Fitbit Charge 5 and will be analyzed via the adherence strategy described above. Validation studies suggest that Fitbit is ideally suited for long-term self-monitoring of PA due to its user-friendliness and extensive use in RCTs [63].

#### Measures of Cardiovascular Outcomes

Cardiovascular outcomes will be indicated by change from baseline to end of 6-month study post intervention in CVD risk score calculated using the 2013 Atherosclerotic Cardiovascular Disease Risk Calculator [35]. The algorithm provides sex- and race-specific estimates for the first CVD events. The scores range from 0% to 100% with higher scores representing poor cardiovascular health status. The composite risk factors included in the algorithm are age, total and HDL cholesterol, systolic blood pressure (including treated or untreated status), diabetes, and current smoking status. These will be measured using the protocols outlined under baseline assessment. The main outcome will be between-group differences in risk score change from baseline. We will use the same strategy to evaluate changes in independent risk factors for CVD (secondary outcomes): waist circumference, weight, BP, Life’s Essential 8, hemoglobin A_1c_ (HbA_1c_), adiponectin, and lipids (total, low-density lipoprotein, and HDL cholesterol).

#### Sample Size Justification

Given our repeated measures study design, we anticipate some participant attrition. To ensure sufficient sample size with complete assessment through 6-months follow-up, we plan to enroll 88 participants (44 per treatment group) retaining at least 76 participants (38 per treatment group) assuming a 14% attrition through the 6-month follow-up based on attrition rates observed in the literature [64]. When estimating within-group changes to describe the effect of PATH on study outcomes using either proportions or means, with at least 38 participants per treatment group (76 total), we would have at least 80% power to detect small-to-medium effect sizes for the efficacy of the PATH intervention. For PA, this corresponds to a mean difference of 20 minutes of MVPA or 13.3% absolute change in terms of adherence to PA guidelines. For CVD risk factors, linear contrasts from either linear or generalized linear mixed modeling will be specified and estimated to compare the treatment groups on the 6-month change in CVD risk scores and risk factors at test wise (Bonferroni-adjusted) significance level of .05.

#### Statistical Analysis

Data will be analyzed using SAS (version 9.4; SAS Institute) to conduct exploratory data analyses for data screening, including missing data assessment, as well as repeated measures modeling, and modeling of attrition. Mplus (version 8.8; Muthén and Muthén) will be used for mediational analyses to explore possible mechanisms of action of the PATH intervention. Data will first be carefully screened with the results of these preliminary analyses informing the final analysis strategies to be applied to address study aims and exploratory analyses. Although hypotheses are stated as directional, hypothesis testing will be nondirectional with the significance level set to .05 and CI estimation at 95%. The randomness of missing data will be investigated using information on participant characteristics to help discern patterns in the missing data, identify possible missing data mechanisms, and inform strategies to handle missing data. If data are not missing at random, we will apply multiple imputations and 2 extreme case scenarios (worst or best case) to address missing data. If nonrandom missingness is suspected, we will use selection or pattern mixture modeling to explore the sensitivity of results to the assumed missing data patterns.

An intent-to-treat (ITT) approach will be used to test the efficacy of the PATH intervention on the distal outcome of adherence to the PA guidelines over time. Since key study end points are assessed at multiple time points, repeated measures modeling (eg, linear or generalized linear mixed modeling methods and or marginal modeling using generalized estimating equations as appropriate, both assuming a normal error structure) will be used to test the efficacy of the PATH intervention on the measures of adherence to the PA guidelines over time (baseline [0 months], and 6 months [long-term] follow-up). To test the hypothesis that the PATH intervention group will have greater adherence to PA guidelines compared with the attention control group, linear contrasts will be specified and estimated to compare adherence to PA guidelines between the treatment groups at 6 months relative to baseline values, respectively. The test wise significance level will be set to .05 (Bonferroni-adjusted). Point and interval (95% CIs) estimates based on linear contrasts will also be computed as both unstandardized and standardized effect sizes of the efficacy of the PATH intervention.

#### Analysis Strategy to Compare the Effects of the PATH Intervention on CVD Risk Factors

A similar repeated measures modeling approach as outlined above will be used to evaluate the efficacy of the PATH intervention compared with the attention control condition on CVD risk factors including CVD risk score, weight, BP, waist circumference, HbA_1c_, and lipids at 6-month follow-up. These measures of CVD risk are either interval or ratio scaled, and a normal error structure (or following a suitable data transformation) will be assumed when modeling. As necessary, generalized linear mixed modeling assuming a binomial error will be applied to model CVD risk factors dichotomized based on clinically meaningful cut points. To test the hypothesis that the PATH intervention group will have a greater reduction in CVD risk score versus the control group, linear contrasts will be specified and estimated to compare the treatment groups on the change in CVD risk scores at 6-months follow-up relative to baseline values at a test wise significance level of .05 and CI estimation at 95%.

### Data Management

#### Overview

All technologies to be used in this study, including the AWS web hosting platform, PATH website, Twilio, SR Fax, ActiGraph GT3X, Fitbit, and Withings apps were reviewed and approved by Emory University’s Enterprise Information Security Team. The entire data collection infrastructure, including the REDCap and PATH platforms, were tested by the study team before it was deployed for data collection. Data will be stored on a password-protected computer with access limited to the staff members who monitor participants’ adherence and safety, and the data management team. Access to the master list of participant names and ID numbers will be limited to the staff members who conduct the screening and intervention procedures as they need to interact with these individuals on a name basis. The staff members who do not interact with participants are blinded to name and treatment assignment. The primary outcomes will be shared in scientific conferences and peer-reviewed publications. Investigators seeking to use the deidentified data will sign a data user agreement with Emory University.

#### Plans to Monitor the Data to Ensure Safety of Participants and Data Integrity

A data safety and monitoring plan will be implemented to ensure the safety of all participants involved in the study and to ensure the validity and integrity of the collected data. The PI and biostatistician (in conjunction with the project coordinators and staff members), will be responsible for the execution of this plan under the oversight of a safety officer and the institutional review board (IRB). The safety officer will act in an advisory capacity to the PI and the National Institutes of Health program director. All adverse events will be documented in REDCap and reported in publications. Major adverse events will be reported to IRB following established protocols. To posttrial care or compensation for harm will be provided. The SPIRIT (Standard Protocol Items: Recommendations for Interventional Trials) checklist is provided in Multimedia Appendix 2.

### Ethical Considerations

The Emory IRB approved the study protocol before beginning the study (IRB approval #STUDY00005168). Written informed consent is obtained from all participants before participation in study procedures. Data is collected using the encrypted and password-protected REDCap and PATH platforms. Identifiable data is only stored in REDCap and is deidentified before being exported for analysis. Confidentiality will be ensured by the use of a simple ID number, which is not encoded to denote other variables. Participants received US $100 compensation and were allowed to keep their Fitbit tracker and Smart Scale for their participation in the study.

## Results

**Figure 2:**
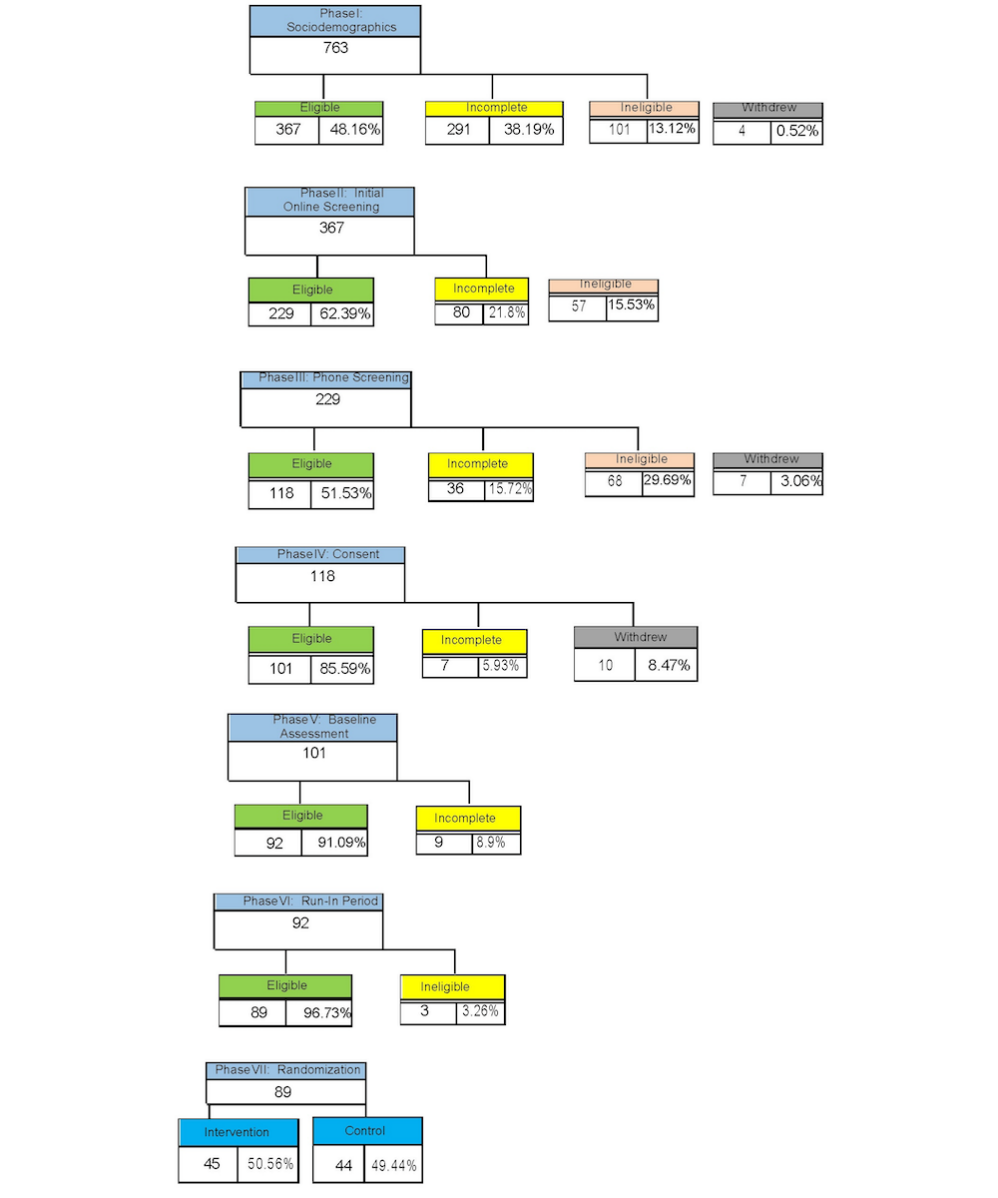
The CONSORT flow diagram.

We screened 763 individuals for eligibility and 89 participants were enrolled and randomized to the intervention (45/504, 50.6%) and control arms (44/504, 49.4%). The average age was 48.7 (SD 12.17) years, and most participants were female (81/504, 90.1%), Black (45/504, 50.6%), and non-Hispanic (83/504, 93.3%). No systematic differences in baseline characteristics were observed between the study arms. The 6-month intervention is currently underway, and the completion of follow-up data collection is expected in February 2025, with results to be published in the Spring of 2025. Figure 2 presents the CONSORT (Consolidated Standards of Reporting Trials) flow diagram with the screening, enrollment, and randomization details.

## Discussion

### Anticipated Findings

The significance of addressing physical inactivity in adults with obesity cannot be overstated. The PATH intervention is testing a novel strategy for addressing the multifaceted barriers to PA among adults with obesity. Our study protocol is grounded in the understanding that traditional PA programs often fail to engage this population because they do not address their unique barriers to PA including stigma, poor fitness, low self-efficacy, unmet weight loss expectations, and limited access to relatable exercise programs [10,15]. By engaging users via tailored workout videos, online coaching, and digital self-monitoring tools, the PATH intervention offers a scalable, participant-centered, and highly accessible solution to address barriers to PA engagement among individuals with obesity. The weight-neutral messaging included in PATH programming is intentionally designed to help participants focus on getting active without being discouraged by limited weight loss progress.

Preliminary evidence from formative studies suggests that the PATH intervention is promising in promoting PA engagement and adherence among adults with obesity [18]. The integration of personalized online coaching with a highly accessible and user-friendly web-based platform addresses key limitations of previous web-based and mobile health (mHealth) PA interventions, which often lacked the individualized support necessary to sustain long-term behavior change [10,14,16]. Our approach in this RCT aligns with that used in recent studies that have highlighted the importance of combining technology with human interaction to promote engagement and adherence in PA programs [14,18]. Moreover, the use of validated smart digital tools (eg, Fitbit tracker, Withings scale, and BP monitor) that are interfaced with the user-facing dashboard on PATH facilitates near–real-time accurate monitoring of multiple cardiovascular health indicators without increasing participant burden. These tools enable the coaches to offer timely feedback and appropriate support during online coaching sessions, helping the participants maintain PA engagement over time.

If successful in promoting PA adherence, the PATH intervention could provide a practical, scalable option that health care providers can recommend to patients who struggle with PA adherence, especially those with obesity. Because PA has been shown to reduce the risk of CVD even without weight loss [8], the PATH intervention has the potential to play a critical role in improving health outcomes in this high-risk population. The program’s weight-neutral messaging encourages the participants to focus on being active with the goal of achieving cardiovascular health benefits, even if no weight loss is attained. This careful messaging is intended to mitigate unmet weight loss expectations, which are a major impediment to long-term adherence to PA in this high-risk population [12].

Understanding the mechanisms of action underlying the efficacy of PATH is highly significant. In this study, we are examining factors such as self-efficacy, social support, and outcome expectations to gain more insights into how the PATH intervention facilitates behavior change. These data will inform further refinement and optimization of the PATH platform. The next phase of optimization could leverage novel digital health technologies such as artificial intelligence for enhanced personalization to further improve the engagement and efficacy of the PATH intervention. This presents a promising opportunity for providing a more tailored and engaging experience for users, which aligns with the growing trend of technology-enhanced preventive health solutions.

This study has a few limitations that are worth acknowledging. The relatively small sample size may limit the generalizability of findings, and our eligibility criteria may favor participants with higher levels of digital literacy and access to technology. Future research should focus on addressing these limitations by conducting larger-scale trials that evaluate long-term efficacy and sustainability of PA adherence, with proactive strategies to reach populations with limited digital access. Our next logical step will be to conduct a full-scale RCT which will be adequately powered to examine if the PATH intervention can increase long-term PA and cardiometabolic health in adults with obesity. After large-scale efficacy trials, comparative effectiveness studies may be needed to determine how PATH performs relative to other web-based, in-person, or hybrid PA interventions to identify the most effective strategies for promoting PA adherence in adults with obesity. In addition, evaluating the cost-effectiveness of PATH compared to traditional PA programs or other web-based or mHealth interventions is also necessary to examine its value as a sustainable solution in real-world settings.

Moreover, future research should explore how the PATH platform can be further tailored to meet the needs of specific subgroups, such as older adults, individuals from diverse cultural backgrounds, or those with comorbidities like diabetes or hypertension. This will enhance the intervention’s relevance and effectiveness for a broader range of individuals. Then, future studies can explore how the PATH intervention can be availed to health care systems to facilitate seamless referral of sedentary high-risk patients by clinicians, making PATH a more practical tool for promoting PA in routine clinical practice. Currently, there are very few options for structured PA programs that health care providers can refer their patients to beyond physical therapy.

### Conclusion

In conclusion, the PATH intervention offers a promising, evidence-based approach to overcoming the barriers that have hindered previous PA programs for adults with obesity. For instance, most of the existing supervised PA programs, such as cardiac rehabilitation are usually short-term and address recovery to a baseline–rather than long-term maintenance of health-enhancing PA. By incorporating user preferences, human support, and a flexible, scalable web-based platform, the PATH intervention is poised to significantly improve PA adherence and reduce the risk of CVD in this high-risk population. If the PATH program proves to be efficacious in promoting PA among individuals with obesity, it could be an option for referral at the end of cardiac rehabilitation to promote and maintain a regular PA regimen. The findings from this RCT will contribute valuable insights to the field of research focusing on behavioral interventions to manage obesity, with potential implications for reducing the burden of obesity-related diseases. This aligns with the broader goal of preventive health care models that emphasize lifestyle modification as a cornerstone for managing chronic diseases [12,65].

## Appendix

Multimedia Appendix 1 The Informed Consent.

Multimedia Appendix 2 The SPIRIT checklist.

Multimedia Appendix 3 Peer-review report from the Lifestyle Change and Behavioral Health Study Section (National Institutes of Health, USA).

## Abbreviations

API: application programming interface
BP: blood pressure
CONSORT: Consolidated Standards of Reporting Trials
CVD: cardiovascular disease
FITT-VP: frequency, intensity, type, time, volume, and progression
HbA1c: hemoglobin A1c
HDL: high-density lipoprotein
HIPAA: Health Insurance Portability and Accountability Act
IL: interleukin
IRB: institutional review board
ITT: intent-to-treat
LDL: low-density lipoprotein
MCP-1: monocyte chemoattractant protein 1
MET: metabolic equivalent
mHealth: mobile health
MVPA: moderate to vigorous physical activity
PA: physical activity
PAR-Q+: PA readiness questionnaire
PATH: Physical Activity for the Heart
PI: principal investigator
RCT: randomized controlled trial
REDCap: Research Electronic Data Capture
RPE: rating of perceived exertion
SPIRIT: Standard Protocol Items: Recommendations for Interventional Trials
TNF-α: tumor necrosis factor alpha
VO2 maximum: maximum rate of oxygen consumption during incremental exercise
