# Supplementary material for: Acceptability and Preliminary Efficacy of a Novel Web-Based Physical Activity for the Heart (PATH) Intervention Designed to Promote Physical Activity in Adults With Obesity: Protocol for a Pilot Randomized Controlled Trial
Source: JMIR Res Protoc. 2025 Mar 18;14:e67972. doi: 10.2196/67972 (PMC11962323; doi:10.2196/67972)
Supplement: Multimedia Appendix 1 [file resprot_v14i1e67972_app1.pdf]

You Are Being Asked to Be in a Research Study

Concise presentation of key concepts

You are being asked to be in a research study. A research study is designed to answer a scientific question. If you agree to be in the study, you will be one of 88 people who are being studied, at Emory.

Why is this study being done?

This study is being done to answer the question: to test if a video-based exercise program can be useful in helping people who have a body mass index of 30* or higher increase their physical activity. You are being asked to be in this research study because there is a need for programs that help people with obesity increase their physical activity. Regular exercise can help reduce the risk of diabetes and heart disease. Recently, our study team developed a web-based "Physical Activity for The Heart" (PATH) program that uses YouTube workout videos to help people increase their physical activity. In this study, we plan to test if our PATH program is helpful in promoting physical activity among individuals with obesity.

Do you have to be in the study?

It is your choice to join this research study. You do not have to be in it. Your choice will not affect your access to medical care for your condition. Before you choose, take time to learn about the study.

What do you have to do if you choose to join this study?

If you qualify and choose to join the study, you will participate for 6 months (3 study visits). The researchers will ask you to do the following:

- We will guide you through measuring your blood pressure, blood sugar levels, blood cholesterol levels, adiponectin, weight, and waist circumference on zoom
- We will ask you to use workout videos to increase your physical activity
- We will ask you to wear a physical activity tracker on your wrist and waist
- You will receive email and/or text reminders to use your selected workout videos
- We will ask you to share your experiences on our online forum
- You will receive a 15-30-minute zoom call from study team every 2 weeks for health coaching or check in. You will also be asked to measure your weight and blood pressure
- At the end of the study, we will ask you to respond to the questionnaires that you did before enrolling in the study and an end of study survey.

ALL of these procedures will be paid for by the study.

How is this study going to help you?

If you are in the study, you will be helping the researchers answer the study question. This study is not intended to benefit you directly. If you manage to increase your physical activity over several days or weeks, you may experience some of the benefits from increasing physical activity. This includes reducing your risk of developing diabetes or heart disease. You need to continue to exercise to sustain this benefit.

What are the risks or discomforts you should know about before deciding?

The study will take time. All studies have some risks. Some risks are relatively small, like being bored or losing time. Some are more serious. Risks for this study include:

- loss of privacy
- breach of confidentiality

You can find a full list of expected risks, their frequency and severity in the section titled "What are the possible risks and discomforts?"

Alternatives to Joining This Study

Since this is not a treatment study, the alternative is not to participate.

Costs

There will be no costs to you for participating in this study, other than basic expenses like transportation. You will not be charged for any of the research activities. There is more information in the "Costs" section further below.

What Should You Do Next?

Read this form, or have it read to you. Make sure the study doctor or study staff explains the study to you. Ask questions such as how much time you will have to spend on the study, any words you do not understand and more details about study procedures. Make sure you understand. Take time to think about this and talk about it with your family and friends.

Emory University
Consent to be a Research Subject

Title: Efficacy of a novel web-based physical activity intervention designed to promote adherence to physical activity guidelines in adults with obesity

IRB #: STUDY00005168

Principal Investigator: Jacob K. Kariuki, PhD, NP
Associate Professor, Emory University Nell Hodgson Woodruff School of Nursing
Phone: 404-727-2353
Email: Jacob.kariuki@emory.edu

Funding Source: National Heart Lung and Blood Institute

Introduction

You are being asked to be in a research study. This form is designed to tell you everything you need to think about before you decide to consent (agree) to be in the study or not to be in the study. **It is entirely your choice. If you decide to take part, you can change your mind later on and withdraw from the research study. You can skip any questions that you do not wish to answer.**

Before making your decision:

- Please carefully read this form or have it read to you
- Please ask questions about anything that is not clear

You can take a copy of this consent form, to keep. Feel free to take your time thinking about whether you would like to participate. By signing this form you will not give up any legal rights.

A description of this clinical trial will be available on http://www.ClinicalTrials.gov, as required by U.S. law. This Web site will not include information that can identify you. At most the Web site will include a summary of the results. You can search this Web site at any time.

What is the purpose of this study?

The purpose of this study is to test if a video-based exercise program can be useful in helping people who have a body mass index of 30* or higher increase their physical activity.

*BMI ≥30 is the medical definition of “obesity.” Many people do not like that word because of the stigma in our society. We only use the term to define who can be in this study.

What will you be asked to do?
You have completed screening questionnaires that show you may be eligible for this study. If those questionnaires indicate that you need to obtain clearance from your doctor, we will need your doctor's clearance to determine your eligibility. Your answers to those questionnaires, your doctor's clearance (if necessary), and the procedures that will be conducted at the Baseline Assessment will all determine if you are eligible.

All research activities are listed as follows:

Baseline Assessment: The Baseline Assessment will take place at a quiet place in your home. The study team will connect with you through a secure Zoom connection to guide you through the assessment, which will take about 90 minutes. The study team will deliver or ship to you all the equipment that you need for the assessment. The baseline assessment will begin by a review of the data that you provided in the questionnaires, after which we will then measure your:

- Blood pressure
- Blood sugar levels collected via finger stick using dry blood spot kit
- Blood cholesterol and adiponectin levels via finger stick using dry blood spot kit
- Weight
- Waist circumference

You cannot join the study if you do not complete the baseline assessment. If you remain eligible after these assessments, we will ask you to wear two physical activity trackers, one on your wrist and another on your waist for 7 days. After that, you will be invited for a one-on-one session where your study group will be assigned. You cannot join the study if you do not wear the trackers as instructed.

Once we are sure you have met all the study requirements, you will be invited for a 30-45-minute Zoom one-on-one session with the study team. If you remain eligible and are willing to participate in the study, we will use a computer program to randomize you into one of two groups. The groups are PATH treatment or Attention control. Randomization means the computer chooses by chance, like a flip of a coin. Neither the study team nor the study participants get to choose their group assignment. Those who enroll in the study will be asked to ship their dry blood spot kits using prepaid envelopes, but to retain the assessment equipment until the end of the study. We ask that if you do not enroll in the study to return all the equipment and samples using a prepaid shipping box we will provide, or by making a pickup arrangement with the study team.

If you are assigned to the Attention control group, you will be provided with:

- An electronic copy of the Attention control booklet that is intended to help you integrate physical activity in your daily life.
- Access to www.health.com, a website written for the public in easy-to-understand language that focuses on general health topics (e.g., dry eye symptoms) and latest medical news.
- Every two weeks, you will receive a 15-30-minute zoom call from the study team to review your progress in the study. The calls may be recorded for quality assurance.
- A monthly newsletter focusing on ways in which you can improve your diet quality.
- At the end of the 6 months, you will be given access to the PATH program without the coaching component.

If you are assigned to the PATH treatment group, you will be given:

- access to the PATH website that includes workout videos that you will be using to help increase your physical activity.
- The study team will use your assessment data to determine the appropriate level for you to start the intervention (e.g., beginner PATH).

- You will access the workout videos in this level using a username and password that will be set up during this session.
- We will also discuss issues related to safety and how to report problems that may arise during the study with you. Every two weeks, you will receive a 15-30-minute zoom call from the study team to review your physical activity routines and set your goal for increasing physical activity. The calls may be recorded for quality assurance.
- A monthly newsletter focusing on ways in which you can improve your diet quality.
- You will also receive automated motivational text/email reminders to do your scheduled workouts (e.g., *It is time for your workout! No matter how slow you go, you're already beating everyone sitting on the couch!*).

Throughout the 6-month Study: We will ask you to self-monitor your daily physical activity. We will ask you to wear a physical activity tracker on your wrist for at least 10 hours per day when you are awake. We will also ask you to measure your weight and blood pressure during the twice/month calls with the study team. The trackers for physical activity, weight and blood pressure will be connected to two apps which will help us monitor your progress throughout the study. A staff member will help you set-up your trackers prior to or during baseline assessment.

6-month Assessment: at 2 weeks and then 1 week before the end of the study, we will remind you (via text message, email, and phone call) to wear the physical activity trackers for 7 days before your end of study assessment. At the end of the study, the waist activity trackers and blood pressure machines need to be returned to the study team. At this Zoom visit, we will repeat all the tests we did at your baseline assessment:

- Blood pressure
- Blood sugar levels
- Blood cholesterol levels
- Adiponectin levels
- Weight
- Waist circumference

We also will ask you to complete the questionnaires that you did before enrolling in the study. We will ask you to complete an end of study survey to share your thoughts and feelings about the study. These will take about 40 minutes.

Adherence Checks: During the study, the study team will review your progress. In addition to random checks, at specified time periods (6, 12 and 18 weeks), the study staff will review your self-monitoring and physical activity data. We may contact you if we detect that you are not wearing the tracker as instructed. You will not have to complete questionnaires or come in for assessment at 6, 12 or 18 weeks.

Who owns your study data and samples?

If you join this study, you will be donating your samples and data. You will not be paid if your samples or data are used to make a new product. If you leave the study, the data and samples that were already collected may be still be used for this study. If you withdraw your consent for the use of your identifiable information, you will also be withdrawn from being in this study. To withdraw your consent for participation in this study, please inform the PI on the first page at the phone, email or address listed. Any identifiable research information that we have collected before you withdrew will continue to be used and protected for confidentiality.

What are the possible risks and discomforts?

There may be side effects from the study procedures that are not known at this time.
The most common risks and discomforts expected in this study are:

<u>Risk of measuring blood sugar and cholesterol and adiponectin levels</u>

During the baseline assessment, we will ask you to prick your finger using a very fine small needle called a lancet. We will ask you to apply 4-5 drops of blood from your finger onto a test kit. This will be shipped to you with other equipment. The procedure may cause minor brief pain (or rarely, bruise) at the point where the blood is taken. The state-of-the-art equipment supplied to you will make the procedure nearly painless. The sample with the dried blood spots will be sent to the lab without identifiers. Only the study team will link your results to you. The sample will only be used to test your blood sugar, cholesterol and adiponectin levels at baseline and 24 weeks. There will be no storage of any blood spots once they are analyzed. We will give you the results, but they will not be entered in your medical record.

<u>Risk of increasing physical activity</u>

Increasing physical activity may lead to muscle soreness. Some soreness is normal, but it should ease over time. You will be provided with a non-slip exercise mat to reduce the risk of slipping during home-based physical activity sessions. Other infrequent risks include feeling dizzy, chest pain or discomfort, and exercise-related cardiac events. During the one-one-one session, you will be advised how to safely engage in physical activity, and when to stop and call for medical help (911 and/or primary care provider) if there is a problem.

<u>Risk of collection of private health information, internet, and text message use, and recording of phone calls</u>

It is always possible that someone who is not involved in this study could access or view your private information that you provided us. We will not place your name on the research data but will instead use an ID code to identify you. It is unlikely that anyone other than limited study staff will be able to link your name to your private information.

Questionnaires completed on the online program will be protected using online data protection measures that are built into the program to prevent data from being released. Information containing your personal information (i.e., consent forms, contact information) will be stored in a separate location from the research data. The research data will be stored without anything that identifies you. Paper-based records are stored in locked filing cabinets in locked offices. Electronic data are stored in password protected databases on a secure server. Access to the research data is limited to members of the research team who have a need to access such information.

Zoom call conversations that will be recorded for quality assurance will be secured in a password protected OneDrive folder. These data will be deleted as soon as they are uploaded on the secure Emory University server. Only the study team will have access to these data.

Although every reasonable effort has been taken, confidentiality during Internet communication, such as doing the surveys or getting text messages, cannot be guaranteed. It is possible that additional information beyond that collected for research purposes may be captured and used by others not associated with this study. To our knowledge, our previous studies using technology have not had this happen and the risk of breach of confidentiality is low.

The less common risks and discomforts expected in this study are:

<u>Risk of wearing physical activity tracker</u>

Wearing a physical activity tracker on your wrist may cause some irritation on the wear location. The waist tracker is worn over cloth using a belt and is not likely to cause irritation.

Rare but possible risks include:
Risk of falling which may occur when exercising in slippery floors or in the context of mental impairment; and risk of heart attack which may occur if the activity levels and intensity are increased rapidly.

Researchers may learn something new during the study that may affect your choice to be in the study. If this happens, they will tell you about it. Then you can choose if you want to stay in this study. You may be asked to sign a new form if you choose to stay in the study.

Will you benefit from the study?

You may not benefit from joining the study. This study is designed to learn more about strategies that can help individuals who struggle with weight issues increase their physical activity. The study results may be used to help others in the future. You may learn things that may help you become more physically active. If you manage to increase your physical activity, you may experience some of the benefits associated with physical activity including reduced risk of diabetes and heart disease.

Will you be paid for your time and effort?

You will receive $20 for completing the baseline and end of study assessments (total =$40). These reimbursements will be done via ClinCard after the completion of baseline and end of study assessments, respectively. You will also receive $60 as reimbursement, via ClinCard, if you use your data plan to access the workouts available on our PATH website. Although we hope that you will commit to do all the workout videos recommended for you each week, you must complete at least 70% of your weekly goal be eligible for this reimbursement. You will receive this reimbursement at the end of the study.

How will your private information be protected?

Whenever possible, a study number, rather than your name, will be used on study records. Your name and other identifying information will not appear when we present or publish the study results.

Certificate of Confidentiality

There is a Certificate of Confidentiality from the National Institutes of Health for this Study. The Certificate of Confidentiality helps us to keep others from learning that you participated in this study. Emory will rely on the Certificate of Confidentiality to refuse to give out study information that identifies you. For example, if Emory received a subpoena for study records, it would not give out information that identifies you.

The Certificate of Confidentiality does not stop you or someone else, like a member of your family, from giving out information about your participation in this study. For example, if you let your insurance company know that you are in this study, and you agree to give the insurance company research information, then the investigator cannot use the Certificate to withhold this information. This means you and your family also need to protect your own privacy.

The Certificate does not stop Emory from making the following disclosures about you:

- Giving state public health officials information about certain infectious diseases,
- Giving law officials information about abuse of a child, elderly person or disabled person.
- Giving out information to prevent harm to you or others.

Giving the study sponsor or funders information about the study, including information for an audit or evaluation.

Storing and Sharing your Information

We will store all the data and blood specimen that you provide using a code. We need this code so that we can keep track of your data over time. This code will not include information that can identify you (identifiers). Specifically, it will not include your name, initials, date of birth, or medical record number. We will keep a file that links this code to your identifiers in a secure location separate from the data.

We will not allow your name and any other fact that might point to you to appear when we present or publish the results of this study.

Your data and specimens may be useful for other research being done by investigators at Emory or elsewhere. We may share the data or specimens, linked by the study code, with other researchers at Emory, or with researchers at other institutions that maintain at least the same level of data security that we maintain at Emory. We will not share the link between the study code and your identity.

We may also place data in public databases accessible to researchers who agree to maintain data confidentiality, if we remove the study code and make sure the data are anonymized to a level that we believe that it is highly unlikely that anyone could identify you. Despite these measures, we cannot guarantee anonymity of your personal data.

We will use your specimens and data only for research. We will not sell them. However, the results of this research might someday lead to the development of products (such as a commercial cell line, a medical or genetic test, a drug, or other commercial product) that could be sold by a company. You will not receive money from the sale of any such product.

Returning Results to Participants/Incidental Findings

Once the study has been completed, we will send each participant a summary of all of the results of the study and what they mean. We will not send individual results unless we find something of urgent medical importance to the participant. The study team will brief the participant on the abnormality detected and refer them to their PCP or free health clinic for further evaluation and management.

Withdrawal from the Study

You have the right to leave a study at any time without penalty.

If you leave the study before the last planned study visit, the researchers may ask you to complete some of the final steps such as lab work or questionnaires as applicable.

The researchers also have the right to take you out of the study without your consent for any reason. They may do this if they believe it is in your best interest or if you do not agree to changes that may be made in the study.

Confidentiality

Certain offices and people other than the researchers may look at study records. Government agencies and Emory employees overseeing proper study conduct may look at your study records. These offices include the Office for Human Research Protections, the funder, the Emory Institutional Review Board, the Emory Office of Compliance. The Study funder may also look at your study records. Emory will keep any research records we create private to the extent we are required to do so by law. A study number rather than your name will be used on study records wherever possible. Your name and other facts that might point to you will not appear when we present this study or publish its results.

People Who will Use/Disclose Your Information:

The following people and groups will use and disclose your information in connection with the research study:

- The Principal Investigator and the research staff will use and disclose your information to conduct the study and give you study related treatment.
- Emory may use and disclose your information to get payment for study related activities and to run normal business operations.
- The Principal Investigator and research staff will share your information with other people and groups to help conduct the study or to provide oversight for the study.

- National Heart Lung and Blood Institute is the Sponsor of the study. The Sponsor may use and disclose your information to make sure the research is done correctly and to collect and analyze the results of the research. The Sponsor may disclose your information to other people and groups like study monitors to help conduct the study or to provide oversight for the study.
- The research team may use and disclose your information, including disclosure to insurance carriers to administer payment for subject injury.
- The following people and groups will use your information to make sure the research is done correctly and safely:
    - Emory offices that are part of the Human Research Participant Protection Program and those that are involved in study administration and billing. These include the Emory IRB, the Emory Research and Healthcare Compliance Offices, and the Emory Office for Clinical Research.
    - Other researchers and centers that are a part of this study.
    - Government agencies that regulate the research including: Office for Human Research Protections.
    - Public health agencies.
    - Research monitors and reviewer.
    - Accreditation agencies.
- Sometimes a Principal Investigator or other researcher moves to a different institution. If this happens, your information may be shared with that new institution and their oversight offices. Information will be shared securely and under a legal agreement to ensure it continues to be used under the terms of this consent.

Contact Information

If you have questions about the study procedures, appointments, research-related injuries or bad reactions, or other questions or concerns about the research or your part in it, contact Jacob Kariuki at 404-727-2353 or Email: Jacob.kariuki@emory.edu

This study has been reviewed by an ethics committee to ensure the protection of research participants. If you have questions about your **rights as a research participant**, or if you have **complaints** about the research or an issue you would rather discuss with someone outside the research team, contact the Emory Institutional Review Board at 404-712-0720 or 877-503-9797 or irb@emory.edu.

To tell the IRB about your experience as a research participant, fill out the Research Participant Survey at https://tinyurl.com/ycewgkke.

Consent

TO BE FILLED OUT BY SUBJECT ONLY

Print your name, **sign**, and **date** below if you choose to be in this research study. You will not give up any of your legal rights by signing this form. We will give you an electronic copy of the signed form to keep.

____
Name of Subject

______________________________________ ________________
Signature of Subject (18 or older and able to consent) **Date** **Time**

TO BE FILLED OUT BY STUDY TEAM ONLY

____
Name of Person Conducting Informed Consent Discussion

______________________________________ ________________
Signature of Person Conducting Informed Consent Discussion **Date** **Time**
