# Supplementary material for: Acceptability and Preliminary Efficacy of a Novel Web-Based Physical Activity for the Heart (PATH) Intervention Designed to Promote Physical Activity in Adults With Obesity: Protocol for a Pilot Randomized Controlled Trial
Source: JMIR Res Protoc. 2025 Mar 18;14:e67972. doi: 10.2196/67972 (PMC11962323; doi:10.2196/67972)
Supplement: Multimedia Appendix 3 [file resprot_v14i1e67972_app3.pdf]

1R01HL164737-01 kariuki, jacob

INCLUSION ACROSS THE LIFESPAN PLAN UNACCEPTABLE
INCLUSION OF MINORITIES PLAN UNACCEPTABLE
INCLUSION OF WOMEN PLAN UNACCEPTABLE
EARLY STAGE INVESTIGATOR
NEW INVESTIGATOR

RESUME AND SUMMARY OF DISCUSSION: The overall goal of this application is to test the efficacy of a web-based intervention, called Physical Activity for The Heart (PATH), for promoting adherence to physical activity (PA) guidelines in a diverse sample of sedentary adults with obesity. During the discussion, the panel noted the significance of targeting inactive adults with a scalable, web-based physical intervention that includes appealing YouTube exercise videos and a coaching platform, thereby, potentially overcoming barriers to PA and increasing uptake. The project builds off strong preliminary data showing good retention and increases in PA and further extends the current project to 12-months with adherence to PA guidelines as the primary outcome. Significance is reduced, however, by the lack of compelling rationale for the sole focus on PA without consideration for other obesity risk factors, such as diet or weight reduction, and thus, the panel judged that the project would only produce an incremental advancement in cardiovascular disease prevention. The project is led by a strong team of investigators, with complementary expertise and a history of collaboration, situated in a conducive environment. Innovation was highlighted with the use of YouTube videos to increase engagement, although, it was not entirely clear how videos would be curated, or whether the intervention would be equally acceptable to men. Although the panel agreed on the rigor of the randomized study design grounded in a theoretical framework, with appropriate outcomes, there were noted weaknesses with the control condition, which was not well-matched to the treatment group. In addition, the panel remarked on weaknesses related to the recruitment plan, exclusion criteria, and a lack of attention to obesity-related comorbidities. Overall, the panel agreed that the application's strengths are offset by its weaknesses and the project's potential impact is moderate.

DESCRIPTION (provided by applicant): Efficacy of a novel web-based physical activity intervention designed to promote adherence to physical activity guidelines in adults with obesity Funding organization: [redacted] PI: Kariuki Jacob, PhD Co-investigators: Lora E. Burke, PhD, MPH; Bethany Gibbs, PhD; Erick Erickson, PhD; Andrea Kriska. Consultants: David Ogutu, BS Project Summary/Abstract The National Guidelines recommend that all American adults achieve ≥150 min of moderate to vigorous intensity physical activity weekly. Yet, adherence to the Guidelines is low, with only 24% of adults attaining the recommended physical activity (PA). The low levels of PA increase the risk cardiometabolic disease, especially among individuals with obesity. Although any increase in PA can reduce the risk, these individuals face complex weight-related impediments including stigma, embarrassment, poor fitness, and low self-efficacy that reduce their engagement in PA. To mitigate these barriers, web-based PA interventions have been developed, but their efficacy is often limited by lack of behavioral coaching and generic content that does not address barriers experienced by those with obesity. Our research team and others have reported that culturally diverse individuals with obesity prefer PA programs that are convenient, fun to engage in, and feature people to whom they can relate especially in body size, fitness level, and age. Yet, there is a paucity of PA interventions intentionally designed to incorporate these preferences. In our prior work, we sought the input of individuals with overweight/obesity to inform the development of a technology-based Physical Activity for The Heart (PATH) intervention that leverages openly accessible platforms, such as YouTube, to provide workout videos that match the specific preferences expressed in our formative studies and the extant literature. We have successfully beta tested the PATH platform and have demonstrated excellent retention,

intervention engagement and preliminary efficacy in our 12-week feasibility studies. In this application, we propose to test the efficacy of PATH in promoting adherence to the PA Guidelines in a diverse sample of inactive adults with obesity. Our long-term goal is to deploy the PATH intervention as a scalable stand-alone program to increase access, reduce time commitment, avoid weight-related stigma, and lessen the impact of unpredictable barriers to PA such as inclement weather or pandemics like COVID-19. We will conduct a 12-month 2-group randomized controlled trial and equally allocate 450 adults to one of the two intervention conditions: Path intervention or attention control. In Aim 1 we will evaluate the efficacy of PATH for improving short- and long-term adherence to the PA Guidelines. In Aim 2 we will compare the effects of the PATH intervention on risk factors for CVD. In the final Aim, we will explore the potential mechanisms of action associated with changes in the PA outcomes. At the end of the study, we will survey user experience and views on the most useful components of the PATH intervention. Our approach is innovative because we leverage freely accessible resources to provide a highly scalable, convenient, fun to engage in PA program that utilizes carefully selected workout videos to help individuals with obesity progress along the PA continuum. This contribution will be significant because PATH could offer a novel, convenient, enjoyable, and highly scalable program that features “similar others” to promote PA in adults with obesity.

PUBLIC HEALTH RELEVANCE: New approaches to promote health-enhancing physical activity in high risk populations are urgently needed. This application seeks to test the efficacy of a novel web-based program that employs remote coaching and culturally salient workout videos to help individuals with obesity adhere to the Physical Activity Guidelines.

CRITIQUE 1

Significance: 5
Investigator(s): 2
Innovation: 3
Approach: 5
Environment: 1

Overall Impact: This application seeks to test the efficacy of an on-line physical activity intervention for individuals with obesity. The intervention involves a web platform that pulls in YouTube exercise videos that the team has identified through preliminary studies will appeal to end users in the intervention. In addition to the YouTube videos, the web platform is able to host a number of other features, such as goal-setting that can be facilitated by a health coach and a personalized user-facing dashboard displaying the users recommended videos as well as progress toward PA goals. The team has provided preliminary data to show good retention at 12 weeks and increased MVPA in comparison to those in a control group. The goal of the proposed project will be to test the effectiveness of the intervention at 6 and 12 months with the primary outcomes being adherence to PA guidelines and secondary outcomes being changes in CVD risk factors. The investigators propose to randomize 450 inactive individuals with obesity to receive the PATH intervention or a control group that receives non-tailored educational information. Many aspects of the approach are strong, but the format may be less appealing to men and it is not clear to what degree a program like this could be useful to individuals with obesity who also have obesity-related comorbidities where increasing PA may be particularly important. Also, while the pilot data is strong and justifies continued testing of the intervention, the significance of the intervention at advancing the field or impacting clinical practice is not clear in the application. A focus only on PA with little discussion of diet or weight reduction for this population may undermine attempts to substantially change CVD risk. The PI is well trained and has experience working with other more senior researchers on the team who are conducting similar work. Given the

balance of strengths and weaknesses the application was viewed as having a moderate to low impact on the field.

1. Significance:

Strengths

- It seems worthwhile to have you-tube style workout interventions that cater to different audiences. The program described appears to be appealing to the target audience.

Weaknesses

- Major: The rationale for focusing only on physical activity versus diet or both (diet and PA) for this population of individuals with obesity was not clearly delineated in the application.
- Major: it is not really clear from the significance section how large of an impact on clinical outcomes or clinical practice a program like this will actually have.
- Major: As outlined, the contribution of this work to the field is understated in the application.

2. Investigator(s):

Strengths

- Dr. Kariuki is an early stage and new investigator and assistant professor in the School of Nursing at the University of Pittsburgh. He has a joint appointment in the Center for Behavioral Health and Smart Technology. He received funding through a diversity supplement to one of Dr. Burke's grants focused on weight loss and he is a co-I on an R35 grant with Dr. Erickson. He has received some small funding form the PRIDE-CVD training program and additional funding from the CTGSA to develop the PATH intervention.
- Dr. Burke is a professor in Nursing and a senior level co-investigator on the project with expertise in weight loss RCTs and the use of technology to enhance weight loss interventions.
- Dr. Erickson a professor in psychology and is an eminent scholar in physical activity and its relation to cognitive outcomes.
- Dr. Gibbs is an epidemiologist who has substantial research experience related to cardiovascular health and disease, especially as it relates to physical activity, CVD assessment, and lifestyle intervention RCTs.
- Dr. Kriska is well-known for her work related to the Diabetes Prevention Project. She is a physical activity epidemiologist with experience developing and testing lifestyle interventions.
- Dr. Sereika is an experienced biostatistician who will assist the team in the analyses proposed for the project.

Weaknesses

- The team is excellent, but there may be a little bit of overlap in what the Co-Is bring to the project, as they have little effort on the project and may be bringing more mentorship than actual work-effort to the project.

3. Innovation:

Strengths

- There is some innovation around the YouTube recommender system to help identify new workouts that a user might enjoy.

Weaknesses

- Many of the aspects highlighted as innovations are fairly standard practice in on-line interventions.

4. Approach:

Strengths

- Pilot data are supportive of their primary aim that focuses on changes in physical activity.
- RCT design with intent-to-treat analytic approach. Power calculations are reasonable. Mediation analysis will be used to examine potential mediators of intervention effects.
- Fidelity assessments among 10% of sample to assess coaching component.
- Objective measurement of physical activity and weight will be used and transmitted to study team via cloud.
- Composite measure of cardiovascular risk which includes age, cholesterol, blood pressure, smoking status, and diabetes, will be used as primary outcome of aim 2.

Weaknesses

- Major: Despite attempts to reach out to men, the format may not appeal to men. It is unclear in the application whether the pilot data support the use of this method for men with obesity.
- Major: the amount of contact that the intervention group receives is a lot greater than the control group and any differences observed could be attributable to attention.
- Major: It is not clear if the intervention is able to impact CVD risk as it will be measured in the larger study, since the pilot data do not show strong effects for blood pressure.
- Exclusion will be anyone with a history of CVD and diabetes. It is unclear how representative this sample will be to individuals with obesity who are also mostly sedentary.
- It is not really clear how new videos are added to the library of culled and reviewed videos or how this works exactly.

5. Environment:

Strengths

- The environment is excellent within the school of nursing as well as the institutional resources available to help launch and manage the study.

Weaknesses

- None noted.

Study Timeline:

Strengths

- Investigators present a 4-year timeline that is justifiable given the scope of the work.

Weaknesses

- None noted.

Protections for Human Subjects:

Acceptable Risks and/or Adequate Protections

Data and Safety Monitoring Plan (Applicable for Clinical Trials Only):

Acceptable

- plan for monitoring is described in detail

Inclusion Plans:

- Sex/Gender: Distribution justified scientifically
- Race/Ethnicity: Distribution justified scientifically
- For NIH-Defined Phase III trials, Plans for valid design and analysis: Not applicable
- Inclusion/Exclusion Based on Age: Distribution not justified scientifically
- The justification for exclusion of individuals over 70 was not scientifically justified but was made because the investigators had not piloted the work in older individuals.

Vertebrate Animals:

Not Applicable (No Vertebrate Animals)

Biohazards:

Not Applicable (No Biohazards)

Resource Sharing Plans:

Not Applicable (No Relevant Resources)

Authentication of Key Biological and/or Chemical Resources:

Not Applicable (No Relevant Resources)

Budget and Period of Support:

Recommend as Requested

CRITIQUE 2

Significance: 2
Investigator(s): 1
Innovation: 1
Approach: 3
Environment: 1

Overall Impact: This application proposes a clinical trial comparing PATH, a technology-based, remotely delivered 12-month physical activity intervention to an attention control group among adults with obesity, with outcomes assessed at 6 and 12 months. The primary outcome is adherence to physical activity guidelines. The study will be led by an ESI with relevant experience and training, and supported by a strong team of experienced co-Is. The research environment is strong. The significance of this proposal is high as a result of an approach that has high potential to overcome barriers to physical activity in adults with obesity and that is remotely delivered and has high potential for scalability. The use of open-source videos and using patient feedback to inform suggestion of videos is an innovative feature of the research. Strengths of the approach include the strong conceptual basis of the intervention, pilot data supporting intervention acceptability and potential for effectiveness, and trial design feature that promote validity and reproducibility (e.g., blinded outcome assessors, treatment fidelity monitoring). Weaknesses are minor to moderate and include a primary outcome of adherence to physical activity guidelines rather than a continuous outcome; absence of a plan to collect qualitative data which may be highly informative given the novelty of the intervention approach; exclusion of individuals with diabetes; and concerns about the feasibility of the recruitment goals. Overall, the study is estimated to have moderate to high impact.

1. Significance:

Strengths

- Increasing physical activity in adults with obesity would have population health benefits.
- Use of open-source videos in the proposed intervention enhances the cost-effectiveness and potential for reach.
- The proposed intervention has good potential to overcomes barriers to exercise in many adults with obesity.
- The remote data collection approach may increase the chances of a representative sample.
- Consideration of past web-based PA interventions in adults with overweight/obesity suggest promise, but there are gaps in the research that this project will fill

Weaknesses

- (minor) The potential for scalability that is inherent in aspects of the intervention (e-health, open source videos) is reduced by use of health coach, although this likely increases the potential for effectiveness.

2. Investigator(s):

Strengths

- The PI, Dr. Kariuki, is an ESI with a PhD in population health and health policy who has relevant training and experience in clinical trials and in prevention of cardiometabolic disease. He developed and pilot tested he proposed intervention.
- The study team includes investigators with expertise in clinical trials (Burke, Kriska), statistics for clinical trials (Sereika), and physical activity (Gibbs, Kriska, Erickson).

Weaknesses

- None noted

3. Innovation:

Strengths

- The study uses existing programs through open-source websites and builds structure around them to promote physical activity and offering advantages in scalability, flexibility for participants.

Weaknesses

- None noted

4. Approach:

Strengths

- The intervention is anchored in a strong conceptual framework and is responsive to barriers to physical activity common in adults with obesity.
- A pilot study of the proposed intervention met recruitment and retention goals and demonstrated acceptability of the program.
- The assessment protocol is thorough and appropriate for study goals, with well-described and previously piloted plans to conduct assessment remotely.
- A control arm mirroring usual care is appropriate for their study aims.
- Steps to enhance validity and reproducibility, including blinded outcome assessment and treatment fidelity monitoring.
- Valid approaches to statistical analyses are planned, using mixed models with intention to treat principles.

Weaknesses

- (moderate) Selection of adherence to PA guidelines, rather than a continuous outcome, for the primary, results in loss of information. Continuous outcomes will be studied as secondary.
- (moderate) exclusion of individuals with diabetes not well justified.
- (moderate) The team appeared to meet recruitment goals in the pilot study, but it isn't clear how quickly they met these goals, making it difficult to evaluate if a goal of 15 participants recruited per month is feasible. The primary care setting is noted as an important recruitment setting for lower income and rural individuals, and it was noted that 10 participants from the pilot were recruited there, but the specific approach that will be employed for this is lacking. It is also noted that the pool who meet BMI criteria from PHN is about 1,000, which seems limited for a study this size.
- (minor to moderate) Given the novelty of aspects of the intervention approach, obtaining qualitative data at the end of the intervention from some participants may be valuable for informing next steps of this research.
- (minor) I don't see any discussion of how the study will address participants' potential interest in weight loss. It seems like many participants who join may be interested in and expect weight loss. This may need to be addressed as part of the recruitment/enrollment process and/or intervention, and assessed.
- (minor) There is reference to an online forum as part of the intervention, which seems like it would be a potentially valuable component, but very limited details are provided.

5. Environment:

Strengths

- The University offers several centers and institutes that add to the intellectual environment and resources available, including a “Hub for Excellence in eHealth Research,” a CTSI, and a Primary Health network

Weaknesses

- None noted

Study Timeline:

Strengths

- The timeline is appropriate for the research plan, though there are some concerns about the feasibility of the recruitment rate.

Weaknesses

- None noted

Protections for Human Subjects:

Acceptable Risks and/or Adequate Protections

- A well-developed plan for protecting human subjects is described

Data and Safety Monitoring Plan (Applicable for Clinical Trials Only):

Acceptable

- The team will form a DSMB

Inclusion Plans:

- Sex/Gender: Distribution not justified scientifically
- Race/Ethnicity: Distribution not justified scientifically
- For NIH-Defined Phase III trials, Plans for valid design and analysis:
- Inclusion/Exclusion Based on Age: Distribution not justified scientifically
- Exclusion of children justified
- Exclusion of adults over 70 because not included in pilot, but this is not a scientific justification.
- Disproportionate enrollment of women not justified scientifically
- Proportion of minorities aimed to enroll (30%) not scientifically justified, and description of what minorities not provided.

Vertebrate Animals:

Not Applicable (No Vertebrate Animals)

Biohazards:

Not Applicable (No Biohazards)

Resource Sharing Plans:

Not Applicable (No Relevant Resources)

Budget and Period of Support:

Recommend as Requested

CRITIQUE 3

Significance: 5
Investigator(s): 2
Innovation: 4
Approach: 4
Environment: 1

Overall Impact: The proposal aims to use a web-based system to increase PA adherence, assess CVD outcomes, and assess theoretical mechanisms of behavior change. This is a team that has experience working together on PA and sedentary reduction interventions. There is expertise in behavior change, PA promotion, and adaptive technology interventions. They are also working with a web-developer who they have a pre-existing relationship. There is also a good amount of preliminary data suggesting that the intervention is feasible and effective in modifying PA. One of the major concerns is the contribution that the study can make as there are similar existing programs. In addition, the behavior change strategies that are targeted appear to be a part of existing interventions. Therefore, the overall impact of the current study to the field of PA promotion in individuals with obesity is judged to be low.

1. Significance:

Strengths

- The investigators identified gaps in the PA promotion in individuals with obesity literature related to web-based approaches not being tailored to individuals with obesity, and using a one-size-fits all, generic approach, all of which the current intervention attempts to address.
- If the aims are achieved, there will be an increase in knowledge related to a web-based PA intervention targeting individuals with obesity.
- Web-based interventions targeting individuals with obesity have been shown to improve PA, so there is a scientific basis for conducting the current study.

Weaknesses

- There is some concern that there is incremental knowledge that will be gained. Relatedly, the alterations to the current program, in terms of visual depictions of similarly sized adults, does not seem to be significant factor in promoting sustained behavior change.
- Fundamental principles for the study include vicarious leaning and individualization. However, these are principles that are incorporated into existing PA behavior change programs, especially since many use Social Cognitive Theory and attempt to build self-efficacy, albeit largely through mastery experiences.

2. Investigator(s):

Strengths

- Members of the investigative team have worked together on various projects. This suggests that the team has the ability to successfully conduct the study.
- The investigator will continue to receive mentoring from Dr. Burke, his current mentor. This relationship seems very stable and productive.
- The expertise needed to conduct the study appears to be present.

Weaknesses

- None noted.

3. Innovation:

Strengths

- The PATH system can recommend videos that are likely to be appealing to the user.

Weaknesses

- None noted.

4. Approach:

Strengths

- There is preliminary qualitative data showing that the idea of a web-based intervention is acceptable to participants.
- The study is theoretically based, and these theoretical concepts are measured for mediation. Therefore, the investigators will be able to determine if these concepts are related to change in the outcome variables.
- The investigators recognize that not all participants will be truly sedentary and therefore, have different goals for those achieving close to 150 min/wk of MVPA, although this is likely to be a very small number of participants.
- There are elements of rigor within the study, including the RCT design, feasibility assessments, statistical analyses, etc.

Weaknesses

- Although 62 of 84 participants have completed the study and some feedback from those who have completed has been received, it seems a bit premature to conduct the current study without feedback from all participants, full feasibility data, and complete outcome data. (moderate)
- Web-use data from the preliminary studies does not show use over time, which is important because the investigators note that other studies have had a drop-off in usage over time and they are attempting to overcome this issue. (moderate)
- It is not clear if all of the videos are within the PATH system or if they are continuously being integrated from YouTube. The criteria for selection of videos is also unclear. (minor)

5. Environment:

Strengths

- The environment at Pitt seems appropriate to conduct the study.
- There is an existing relationship with the web developer who conduct all of the web-based programming.

Weaknesses

- None noted.

Study Timeline:

Strengths

- Appropriate

Weaknesses

- None noted.

Protections for Human Subjects:

Acceptable Risks and/or Adequate Protections

Data and Safety Monitoring Plan (Applicable for Clinical Trials Only):

Acceptable

- o More detail on the roles and responsibilities of the DSMB would be helpful.

Inclusion Plans:

- Sex/Gender: Distribution justified scientifically
- Race/Ethnicity: Distribution justified scientifically
- For NIH-Defined Phase III trials, Plans for valid design and analysis: Not applicable
- Inclusion/Exclusion Based on Age: Distribution not justified scientifically
- A stronger case for the upper age exclusion is needed. The fact that the intervention has not been used in the upper age range does not say why this specifically is a reason to exclude them.

Vertebrate Animals:

Not Applicable (No Vertebrate Animals)

Biohazards:

Not Applicable (No Biohazards)

Resource Sharing Plans:

Unacceptable

- No plan was provided

Authentication of Key Biological and/or Chemical Resources:

Acceptable

Budget and Period of Support:

Recommend as Requested

THE FOLLOWING SECTIONS WERE PREPARED BY THE SCIENTIFIC REVIEW OFFICER TO SUMMARIZE THE OUTCOME OF DISCUSSIONS OF THE REVIEW COMMITTEE, OR REVIEWERS' WRITTEN CRITIQUES, ON THE FOLLOWING ISSUES:

PROTECTION OF HUMAN SUBJECTS: ACCEPTABLE

INCLUSION OF WOMEN PLAN: UNACCEPTABLE

The disproportionate enrollment of women was not scientifically justified.

INCLUSION OF MINORITIES PLAN: UNACCEPTABLE

The distribution of minorities and enrollment targets were not scientifically justified.

INCLUSION ACROSS THE LIFESPAN PLAN: UNACCEPTABLE

There was inadequate scientific justification for the exclusion of participants over 70-years-old.

COMMITTEE BUDGET RECOMMENDATIONS: The budget was recommended as requested.

Footnotes for 1 R01 HL164737-01; PI Name: kariuki, jacob kigo

NIH has modified its policy regarding the receipt of resubmissions (amended applications).See Guide Notice NOT-OD-18-197 at https://grants.nih.gov/grants/guide/notice-files/NOT-OD-18-197.html. The impact/priority score is calculated after discussion of an application by averaging the overall scores (1-9) given by all voting reviewers on the committee and multiplying by 10. The criterion scores are submitted prior to the meeting by the individual reviewers assigned to an application, and are not discussed specifically at the review meeting or calculated into the overall impact score. Some applications also receive a percentile ranking. For details on the review process, see http://grants.nih.gov/grants/peer_review_process.htm#scoring.
